# Rupture of the Stomach, Ect.

**Published:** 1844-06-01

**Authors:** John McCreery

**Affiliations:** Somerset, Pa.


### THE MEDICAL EXAMINER,

### And Record of Medical Science.

Vol. VII.] PHILADELPHIA, SATURDAY, JUNE 1, 1844. [No. 11.

### RUPTURE OF THE STOMACH, ECT.

*Trial of Charles Hoyle, for the murder of Jacob Miller.*

[Reported for the Examiner, by John McCreery, M. D., of Somerset, Pa.]

Charles Hoyle was tried at April sessions of Somerset County, Pennsylvania, for the murder of Jacob Miller, on the 13th January last. It appeared that on the evening of that day, Hoyle in company with two others was returning from town in a state of partial intoxication. On the way they were overtaken by Miller, between whom and Hoyle there was an old grudge. A quarrel ensued, and H. struck Miller once or twice about the head, knocking him against a fence, but not felling him. He immediately recovered himself and proceeded on his way, Hoyle following and kicking him several times about the legs. It was now after night, and dark. Miller went on about half a mile, and stopped at the house of a brother-in-law of Hoyle. He said that H. had struck him. When examined, no injury was found upon his person. He soon started towards home, and some time after was found about two miles farther on, lying on his face upon the ground, and unable to walk. When carried into the house adjoining he complained very much of his head, was sick and faint; and made several ineffectual attempts to vomit during the night. A considerable tumour was discovered behind the left ear, and blood flowed from his ears and nose. At this time he appeared to be perfectly sensible, and continued so the next day, during which he was hauled home, a distance of a mile and a half, was bled and took some medicine. On the second and third days, however, he was delirious, but on the fourth day he was again sensible, and died the same evening, 96 hours after the affray. Three days afterwards an inquest was held upon the body; Hoyle in the meantime, had escaped, but was pursued and taken.

A *post mortem* examination was made by Drs. McCreery and Berkey, 60 hours after death. There were no signs of putridity about the body. It appeared to be that of a young man, some 20 years of age, of small stature, slender, pale and delicate, and of a strongly marked scrofulous habit. There were no external marks of violence upon the body except a slight abrasion behind the left ear, and one or two about the face. Two sinuses were observed upon the left hip which were supposed to indicate the existence of morbus coxarius, and attention was first directed to this part. The joint was found completely anchylosed, with great enlargement of the head of the femur, the shaft being carious so that a probe passed for four or five inches longitudinally through the bone. The disease was evidently chronic and quiescent, exhibiting no marks of recent inflammation.

Upon exposing the brain, its vessels were found very much engorged with dark venous blood—none of them ruptured however; about two ounces of colorless serum in the ventricles—membranes natural—no softening of the substance of the brain. The viscera of the thorax natural and healthy in appearance—no tuberculization of the lungs.

In the cavity of the abdomen there was a large effusion of *fluid* blood, mixed with the contents of the stomach, consisting of bile and the ordinary fluid of that organ, which were found to have escaped through a solution of continuity of some three to three and a half inches in extent, in the situation and in the direction of its lesser curvature, commencing half an inch from the pyloric orifice, and extending along the attachment of the omentum minus towards the cardia. The edges were straight, but not smooth. The stomach was collapsed and empty—its mucous membrane pale and somewhat softened, but presenting a remarkable arborescence of its vessels, which were enlarged, some to the size of a crow quill, and all distended with coagulated blood. This was more particularly the case towards the smaller extremity. There was also an extensive ecchymosis beneath the mucous membrance on the anterior face of the stomach. The spleen was very much engorged, and softened to a consistence scarcely greater than that of coagulated blood—a mere pulp. The liver too was somewhat engorged, but not conspicuously; the other organs natural. *There was not the slightest appearance of contusion upon the abdomen externally.* This was the case of the commonwealth, as it went to the jury.

For the defence, it was submitted that the prosecution had failed to connect the *post mortem* appearances of the body with the assault of Hoyle; it was urged as extremely improbable, if not impossible, that a man should be able to walk a distance of two miles after a rupture of the stomach inflicted by a blow—or that he should live four days afterwards. It was proved that near where he was found there was ice upon the road, and from the blood and other appearances on it, it was inferred he had fallen upon it, and had been injured on the rough projecting stones which were also there. It was argued also that this was the more likely, as he was crippled by the disease of his hip, and was consequently unable to walk steadily. In about twenty minutes after retiring, the jury brought in a verdict of "*not guilty*."

It is proper to observe here, that the declarations of the deceased, which were much relied on by the commonwealth for the conviction of Hoyle, were not admitted in evidence by the court; it was however given before the inquest. Miller alledged at different times, that H. had returned and assailed him again at the place were the appearances of blood, &c., were found on the road; that he had knocked him down and jumped upon him, and had thus inflicted mortal injury upon him. It was, however, testified upon the trial by both the persons who were with Hoyle, that he continued with them a distance of three miles beyond where Miller said he attacked him the second time; and nearly to his own house.

Now what was the cause of death in this case? Was the stomach really ruptured by the violence inflicted by H. at any time, or was it done by the fall upon the ice and stones, or was it done in any way during life? And if done after death, how? It is quite possible that the stomach might be ruptured by a blow on the epigastrium, and yet no marks be found upon the abdomen, but it is certainly contrary to all received opinions that a man should live for four days afterwards, with a rupture of this extent; the case of St. Martin to the contrary notwithstanding. But supposing the stomach were not ruptured during life. Then what caused the congestion of the brain, and what agency had it in causing death? Was it the result of violence directly to the head, or of the condition of the stomach as evidenced by the pathological appearances, independently of the rupture? whether those were caused by chronic disease or recent injury? Or was its occurrence entirely incidental, and unconnected with the injuries? We confess ourselves unable to answer these questions to our own satisfaction, from the imperfect history of the symptoms, and of the case, subsequent to the reception of the injury, and in hope that some of those who are familiar with such investigations will throw some light upon it. The case is a curious one, and important in a medico-legal point of view. It will be important, should it be decided that the stomach was ruptured in any way during life, and it will be perhaps not less so, should it be found that it was done after death, without anything having occurred in either case which could lead us to suspect such a lesion.

The above report is interesting in several points, but its value is very much lessened by the imperfection of the evidence, and especially as to the symptoms under which Miller laboured during the time which elapsed after he was beaten, until his death. From Dr. McCreery we learn that he was attended during that time by an "*Indian Doctor*," one who was, of course, wholly incapable of appreciating the symptoms, or of giving any rational account of the case. Dr. McCreery also informs us, that he "was perfectly temperate in his habits, and had not drunk a drop of spirits on the day of the affray." It appears to us very manifest, that Miller died from the abuse he received on that occasion, the verdict of the jury to the contrary notwithstanding. Nothing is said of his being sick prior to the affray. He was struck "once or twice about the head," and knocked "against the fence," and "kicked several times about the legs"—but it is quite likely, from what happened afterwards, that some of the kicks extended higher up, without being observed by the witnesses, as "it was now after night, and dark." The immediate cause of his death was, most probably, the injured condition of the stomach. "Softening of its mucous membrane," "ecchymosis," "remarkable arborescence of its vessels, which were enlarged, some to the size of a crow-quill, and all distended with coagulated blood," indicate clearly enough that inflammation had existed, and the rupture that followed may be regarded as the result of the softening caused by the inflammation. When found, after the beating, he was "lying on his face upon the ground, and unable to walk"—and it appears further more, that he "made several ineffectual attempts to vomit during the night"—which would seem to show, that the stomach had been injured at the time, although it might have been only symptomatic of injury of the brain.—Editor.

